# 'Breaking the cycle': a qualitative study exploring general practitioners’ views of infant mental health

**DOI:** 10.3399/BJGPO.2023.0009

**Published:** 2023-09-06

**Authors:** Anna De Natale, Sophie Hall, Anne McFadyen, Helen Minnis, David N Blane

**Affiliations:** 1 School of Medicine, Dentistry & Nursing, University of Glasgow, Glasgow, UK; 2 CAMHS, NHS Lanarkshire, Glasgow, UK; 3 Infant Mental Health Clinical Adviser, Scottish Government Perinatal and Infant Mental Health Advisory Group, Edinburgh, UK; 4 Mental Health & Wellbeing, School of Health and Wellbeing, University of Glasgow, Glasgow, UK; 5 General Practice and Primary Care, School of Health and Wellbeing, University of Glasgow, Glasgow, UK

**Keywords:** infant health, mental health, general practice, adverse childhood experiences, socioeconomic factors, qualitative research

## Abstract

**Background:**

Infants living in areas of socioeconomic deprivation are more likely to have adverse childhood experiences (ACEs), which are associated with infant mental health (IMH) problems and poor physical and mental health outcomes throughout the life course. As part of the development of IMH services in Scotland, studies are being conducted to explore various stakeholders’ perspectives.

**Aim:**

To understand the views and experiences of GPs working in socioeconomically deprived areas in relation to IMH.

**Design & setting:**

Qualitative study with GPs working in deprived urban communities in Scotland, UK.

**Method:**

Semi-structured interviews were conducted with 12 GPs from 11 practices. Transcribed interviews were thematically analysed, following the Braun and Clarke framework, using NVivo (version 12) software.

**Results:**

The following three overarching themes are presented: (1) Deep End GPs’ inherent understanding of IMH, owing to their placement in deprived communities and their under-recognised role in current IMH provision; (2) Factors influencing how communities might perceive IMH, including the potential associations of IMH with parental blame or judgement in areas of socioeconomic deprivation; and (3) Using previous experience to visualise future IMH service delivery, particularly improving on current shortcomings of connectivity and accessibility of services, to develop successful new services.

**Conclusion:**

GPs in areas of socioeconomic deprivation have a deep understanding of the issues affecting IMH, although do not necessarily relate to the term ‘IMH’. New community-based IMH services are much needed, particularly in deprived areas. However, the pre-existing role of primary care must be recognised, supported, and integrated into new services, alongside training to increase IMH awareness among GPs and other primary healthcare professionals (HCPs).

## How this fits in

Infant mental health (IMH) services are being developed in the UK and internationally. Few studies have explored GPs' views and understanding of IMH. In this qualitative study, GPs in deprived areas — where IMH needs are greatest — reported a deep understanding of the issues affecting IMH, although do not necessarily relate to the term 'IMH'. There is a need for targeted IMH training, and involvement of primary care and patient perspectives in the development and integration of new IMH services within existing systems.

## Introduction

Infancy is considered to span from conception to 3 years or even 5 years of age, and infant mental health (IMH) describes the '*capacity to experience, regulate, and express emotions, form close and secure relationships, and explore the environment and learn*'.^1^ Infant–caregiver relationships play a pivotal role in infants’ social and cognitive development.^2–5^

Stressful early experiences, known as ACEs,^6^ are associated with mental and physical health issues during infancy and throughout the life course.^7–10^ Infants and children living in areas of socioeconomic disadvantage are more likely to have a higher number of ACEs.^11–13^ Developmental problems, such as attention deficit hyperactivity disorder (ADHD) and autism, can also impact on IMH^14,15^ and disproportionately affect families in deprived areas owing to their increased prevalence and lower rates of diagnosis.^16–20^

The Scottish Deep End Project is a network of GPs from the most socioeconomically deprived practices in Scotland,^21^ which advocates for local and national action to reduce health inequalities.^22,23^ As such, the Scottish Deep End Project has published reports and set up initiatives, such as the Govan Social and Health Integration Partnership (SHIP) project, which address the health needs of vulnerable children and families.^24,25^

In 2019, the Scottish Perinatal and Infant Mental Health Programme Board^26^ was set up to implement IMH services in NHS Scotland. In 2020, the first IMH services were established in Fife and Lanarkshire.^27^ Shortly before this study was conducted, Glasgow established an IMH service taking referrals from health visitors (HV), family nurses, and midwives where there was concern for the infant’s development or the infant–caregiver relationship.^28^

As part of the continued development of IMH services in NHS Greater Glasgow and Clyde (NHSGGC), the aim of this qualitative study was to understand the perspectives of Deep End GPs in relation to IMH. The research questions were as follows:

1. How do GPs in areas of socioeconomic deprivation understand IMH?
2. How do GPs believe the communities and other members of the primary healthcare (PHC) team perceive and engage with IMH?
3. What are GPs’ views of the proposed new IMH service?

## Method

Qualitative semi-structured interviews were conducted to explore GPs’ views on IMH.

### Participants and recruitment

A purposive sample of GPs working in Deep End practices was used with a recruitment target of 12 participants.

The Deep End Steering Group was informed of the research at their meeting in December 2021 followed by a formal email invitation to participate in January 2022. The Deep End Steering Group consists of approximately 40 GPs, across more than 35 practices, with 25 of these GPs working in practices in NHSGGC. Although all members of the steering group were contacted, initially only those based in NHSGGC were invited to take part. A follow-up email invitation was sent to increase numbers and reach the target of 12 participants. Interested GPs were then provided with more information. There were no incentives to participate.

Written and verbal consent was obtained before interviews. There were no pre-existing relationships between the researchers conducting interviews (ADN and SH) and the participants.

### Data collection

**Figure 1.**
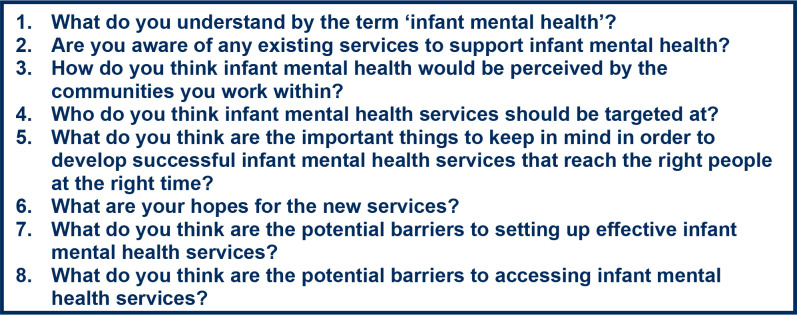
The eight main prompt questions for the semi-structured interviews

All interviews were conducted (between February and October 2022) by a female BMedSci student (ADN), with a female senior child and adolescent psychiatry trainee (SH) co-conducting three of the interviews. Interviews were conducted remotely using Microsoft Teams, Zoom, or phone and were audio-recorded. The interview duration ranged between 30 and 65 minutes, with a mean duration of 41 minutes. A topic guide identified key areas to explore, with the main prompts shown in Figure 1. These main prompt questions were developed by the research team, which included a GP (DNB). They were adapted from previous qualitative research conducted by the team.^29^ No changes were made to the prompt questions during the process of data collection. Demographic data were collected from the participants.

This study was conducted and reported in line with the consolidated criteria for reporting qualitative research (COREQ).^30^

### Analysis

**Figure 2.**
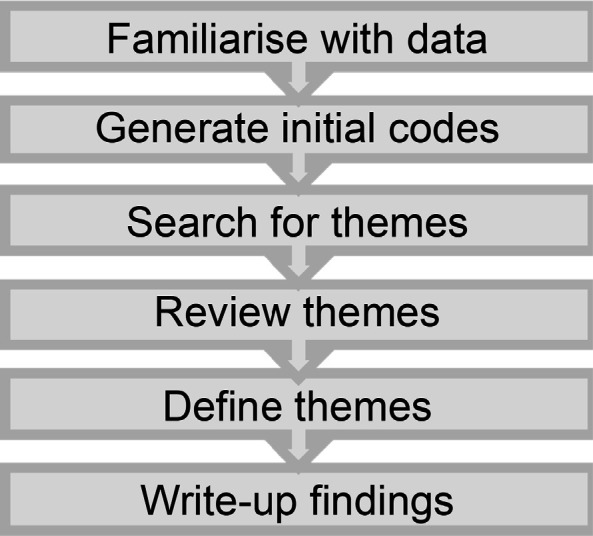
A flowchart showing the stages of Braun and Clarke framework for reflexive thematic analysis^31^

Audio-recordings were transcribed verbatim, anonymised, and analysed inductively. Reflexive thematic analysis (RTA) was carried out by ADN using the Braun and Clarke framework^31^ (Figure 2). Coding was done by familiarising with the transcript and identifying ‘labels’ for points discussed. NVivo (version 12) software was used to assist with this process. An example of codes with illustrative quotes is shown in Table 1. Independent coding of four transcripts by DNB and SH was performed and a coding framework was then developed after discussion within the research team. Consensus was reached through discussion and the coding framework was then applied to subsequent transcripts by ADN, before the development of themes. Codes were then grouped into themes and sub-themes.

**Table 1.** An example of codes with illustrative quotes

Code	Quotes
Participant unfamiliar with IMH	*'... isn't a term I'd come across before.'* *' ... concept of the infant's mental health ... that's* [never] *come up.'*
Focus on physical health of infant	*'... when we talk about infants it's usually about their physical health needs.'* *'I don't think I'm thinking about the infant's mental health.'*
Many parents approach GP with infant concerns easily	*'... quite quick to come and say something is not right.'*
Lack of existing NHS services for IMH	*'I can't think of any other sort of NHS service off the top of my head.'*
If aim of service was understood, parents would be eager to engage	*'... if the term IMH was seen as helping those sort of things ... I think patients would be keen for it.'*
General MH understanding and acceptance of support is good in community	*'... patients understand that and actually take that transition I think quite well.'* *'I don't think particularly people shy away from presenting to services about their mental health.'*
Integration with PNMH	*'I was wondering, you know, the new service, you know, first hearing about it, how it would overlap with perinatal health services.'*

IMH = infant mental health. MH = mental health. PNMH = perinatal mental health.

### Reflexivity

Before data collection, the research team conducted semi-structured interviews among themselves about IMH to explore the positionality of the researchers owing to the subjectivity of RTA.^32^ Audio-recordings were reviewed between interviews to examine the questioning approach for any bias that might have influenced discussion.^33^

## Results

Twelve GPs from 11 practices were interviewed (see Table 2). The initial 10 participants were recruited on the basis that they worked in NHSGGC. However, after the second email invitation, it was agreed that two GPs currently working outside NHSGGC could be recruited, after expressing an interest in taking part in the study. There was very little variance among participants in relation to the main themes presented.

**Table 2.** Participant characteristics

Participant ID	Age group, years	Years in practice	Percentage of practice patients living in datazones defined as the 15% most deprived^^45^^
M1	30–39	<5	50–65
M2	30–39	5–10	66–80
F1	50–59	21–25	50–65
F2	50–59	>25	50–65
F3	40–49	5–10	66–80
F4	30–39	5–10	>80
F5	50–59	>25	66–80
F6	60–69	>25	66–80
F7	40–49	16–20	36–49
F8	50–59	>25	50–65
F9	50–59	>25	30–35/66–80^a^
F10	40–49	11–15	50–65

^a^F9 works in two different practices.

The following three main themes were derived (see Table 3):

1. Deep End GPs’ inherent understanding of IMH;
2. Factors influencing how communities might perceive IMH; and
3. Using previous experience to visualise future IMH service delivery.

**Table 3.** Main themes and sub-themes

Main themes	Sub-themes
Deep End GPs’ inherent understanding of IMH	Understanding of IMH and its contributing factors.Views and experiences of PHC's current role in IMH.Personal experience as parents.
Factors influencing how communities might perceive IMH	Community engagement with PHC.Community reception to enquiry about general mental health.Community understanding of IMH.
Using previous experience to visualise future IMH service delivery	Views on current services.Views on the role of new IMH services.Views on the opportunities and challenges for a new IMH service

IMH = infant mental health. PHC = primary health care.

The findings are supported by illustrative data. Extracts used are identified by participant ID, for example, M1.

### Deep End GPs’ inherent understanding of IMH

#### Understanding of IMH and its contributing factors

While initially some participants expressed their unfamiliarity with the term IMH, others felt that their experience as a Deep End GP, or in some cases their areas of special interest, gave them a clear understanding of the topic:

'*My experience is that we've got a huge understanding … we’re the ones that are seeing the cycle.*' (F3)

One participant expressed how differently mental health manifests in infants compared with older children or adults, and three highlighted how the non-verbal aspect of infancy leaves them overlooked. GPs shared a holistic understanding of IMH and how the wider environment influences infants. Several referred to perpetual family cycles of ACEs and mental health issues they see in Deep End practices, and how the caregiver’s own upbringing influences interactions with their infant:

*'We’ve also got second- or even third-generation parents who themselves had no parenting, no nurturing parenting, so they have no idea at all what to do.'* (F6)

#### Personal experience as parents

Two participants referenced their personal experiences as parents in their understanding of IMH and service delivery. Another, without children, discussed how being a parent could influence awareness of IMH:

*'A lot of that sometimes is reassuring* [the parent]*, but I sometimes say that, and then I think, "I haven’t had children, so I’m not sure".*' (M1)

#### Views and experiences of PHC’s current role in IMH

Participants emphasised the central role that primary care plays in identifying and supporting vulnerable families. This was attributed to the unique and longstanding placement of GPs within the community and their multigenerational relationships with families:

*'GPs are in a very privileged position because we have often three generations of families as patients. So, if you’ve been a GP for a long time, people like that local knowledge and you get to know that family dynamic very well, so families will often come to you.'* (F5)

Some participants thought GP work on infant wellbeing was underrecognised by other parts of the health service, believing an infant focus, particularly in the Deep End, already exists in everyday general practice and in developing new programmes:

*'What you're relying on is … an intuition and a skill and experience that’s not measured, not appreciated, not necessarily understood by the wider health system.'* (F3)

Conversely, some participants felt their practice was not infant-centred, focusing more on the mother or other adults. Two participants expressed the challenge of separating IMH from maternal mental health. When practice was infant-centred, it concentrated on physical wellbeing, monitoring clinical signs of development; for example, head circumference and weight measurements. Participants associated this with the lack of IMH training for GPs to identify IMH issues and confidently communicate concerns to caregivers.

Many expressed the challenges of time and resource constraints, discussing how practice has changed during their careers, as the service has become less holistic and opportunities to establish trusting relationships with families have reduced:

*'When I was a trainee, you did a maternity clinic in the GP surgery and everybody came in and it was great, and it wasn’t just about measuring the bump, mum’s tummy, it was about, "how is home?", you know, "have you got money?"… you had a relationship with everybody, and mums knew you and they trusted you and they told you things.'* (F1)

One participant discussed how the COVID-19 pandemic has caused the loss of the 'art of general practice' and disrupted continuity of care.

When considering IMH, participants saw themselves as part of a 'triad' with HVs and midwives. Some identified fragmentation of the triad, attributing this to COVID-19 and recent changes to service delivery, notably health visiting. Positive and negative experiences of communication with HVs were expressed. However, the value of HVs was highlighted by all participants, noting their integration within the community, supportive relationships with families, and key role in identifying vulnerable families.

### Factors influencing how communities might perceive IMH

#### Community engagement with PHC

Participants believed that parents felt comfortable approaching the GP, HV, or midwife with any concerns. However, several GPs referred to 'the unknowns', who slip under the radar:

*'*[COVID-19] *has probably made it really difficult because a lot of stuff, kids would come with their parents to the surgery, and you just maybe pick something up when the mum or dad were in … So, I think, kids have kind of just been hidden away and, you know, we’ve not really seen them as much.'* (F7)

#### Community reception to enquiry about general mental health

Participants believed that patients openly discussed their mental health, accepting it as a normal part of GP consultations:

*'Patients now will phone up and say, "it’s my mental health doctor"… it’s much more of a kind of recognised thing now, whereas that would never really have happened in the past and you would have been sort of teasing things out of people.'* (F2)

This was attributed to increased societal discourse around mental health. Two GPs reflected on the resulting blurred lines between normal emotional reactions and mental health issues:

*'Part of the problem with destigmatising mental health is that what we've done is we've made finding everything difficult in life a mental health problem, when actually it’s a distress problem …*' (F3)

#### Community understanding of IMH

The concept of IMH was perceived as important to parents, with one participant noting that child mental health and neurodiversity is becoming more accepted in society. Another participant indicated that parents have an intuitive understanding, but all thought that the term ‘*infant mental health*’ would be unclear to parents. However, if the aim of the service was understood, it would be well received. Despite this, some also discussed the potential stigma for parents, possibly perceiving IMH interventions as parental failure. One participant highlighted the impact of IMH stigma on families on top of the pre-existing stigma that already marginalises them owing to their socioeconomic status:

*'We’re talking about this label of infant mental health, and me as a health professional doesn’t really know what that means, then what is a new family’s perception of what that means … if you are labelling their child as having mental health problems when they are a month-old, the mum wouldn’t take too well to that.'* (M1)

Some participants, therefore, suggested naming the service differently, distinguishing its role from Child and Adolescent Mental Health Services (CAMHS) and adult mental health services. Three participants suggested ‘Infant Wellbeing Service’ would sound more holistic and less stigmatising.

### Using previous experience to visualise future IMH service delivery

#### Views on current services

Participants expressed challenges accessing CAMHS or social work support, with intervention occurring only at the severe end of the spectrum. One participant discussed how serious exacerbations of mental health problems could be mitigated with earlier intervention. Another participant highlighted that Deep End communities associate social work with drastic actions, such as removing children, owing to their high threshold for intervention. The exclusionary criteria for mental health service referral were considered by one GP as making already marginalised patients feel more disenfranchised.

All participants relayed the challenges Deep End patients face accessing health services, including geographical barriers, which make attending non-local appointments or support groups impossible. Some thought other parts of the health service were unaware of these logistical barriers.

*'Health services are very good at setting themselves up to suit the service, but not to fit the patient, and patients don't fit into pigeonholes, and that’s where in general practice we often find problems.'* (F8)

The valuable contribution of third-sector organisations supporting child wellbeing was emphasised. These organisations were recognised for skilfully embedding into communities and holistically supporting the family. One participant noted how these organisations were more successful at achieving community-based goals compared with central NHS management. Another participant expressed concern at how the activities of some organisations might disenable parents, while being unsustainable owing to potential instability of third-sector funding.

Participants described poor communication with secondary mental health services, schools, and nurseries. One participant raised the lack of mutual understanding at the primary–secondary care interface. In contrast to secondary care, another participant mentioned the successful communication with well-integrated community mental health services. The Govan SHIP project^34^ was provided as an example of a project where good communication with a multitude of services was at the centre of successful service development.

In addition to HVs, the valuable work of other healthcare professionals (HCPs) in current IMH provision, such as family nurse practitioners and community link workers, was highlighted.

Participants described a lack of continuity after perinatal mental health (PNMH) services, and an absence of support for children too young for CAMHS. As GPs, they identified the need for one dedicated service to refer families into:

*'I despair of a proper infant mental health service who have a proper understanding and willingness to address this very basic, but hugely important issue, and when I say, "despair", I mean despair.'* (F6)

#### Views on the role of new IMH services

Participants thought IMH services should be community-based, universally accessible, and developed with the most vulnerable service users in mind:

*'Let’s do things differently moving forward, and let’s look at less siloed, less super specialist, less exclusive models, and models that are … community-based working across different sectors, accessible to wider primary care teams … and just really think about how we can do things differently proportionate to need.'* (F10)

There was agreement that services should be local, thereby avoiding geographical barriers. One participant suggested that delivery should be adaptable to local pre-existing services and needs. To maximise accessibility and flexibility, some thought it should be a combination of drop-in and appointments via self-referral or referral from HCPs:

*'The reality is in these services you have a huge number of* [non-attendance at appointments]*, so you could develop a hybrid service where you allow an element of drop-in.'* (F6)

Others believed that a self-referral system could overload the service and expressed the importance for primary care to maintain a 'gatekeeper role'.

Participants saw clear referral pathways and training for professionals as vital, to improve HCP accessibility:

*'There’s a real need for a streamlined pathway to help struggling families, whether it’s to get a formal diagnosis of something or whether it’s just to be told that your child doesn't have these but does have other behavioural issues and we will work with you as a family to help, but it would be nice to have a one-stop shop.'* (F9)

One participant discussed the 'hub-and-spoke' model for increasing accessibility to the service:

*'Even if it was based in somewhere like the children’s hospital or wherever else, that maybe if there were spokes as it were like community kind of outreach clinics in each end … one in the Southside, once a month, one in the East End … that kind of thing, I think … that would make it more likely for people to engage.*' (M2)

Three participants stressed the importance of the new service building on pre-existing work as opposed to starting from scratch. One GP felt that pressure on GPs would be alleviated by shifting current tasks to the new service.

Participants highlighted the importance of community education to raise awareness and convey developments in scientific understanding to the wider public:

*'I think child development should be a universal concern, so you want all mums and dads and new infants … progressing well, and to be supported, so you embed it in universal services, that’s non-stigmatising as well, but it requires a bit of education to fix that.'* (F5)

Several participants believed IMH teaching should start at school, raising awareness from the most fundamental level.

It was widely believed that the service should take a holistic approach, supporting the infant and wider family, and therefore should be multi-agency. Although participants thought it should be a universal service regardless of background, some believed that it should particularly target areas of socioeconomic deprivation and identify families with mental health and addiction issues, or chronic illness, and parents who have used PNMH services.

#### Views on the opportunities and challenges for a new IMH service

The following seven key challenges highlighted by participants are outlined below:

1. Poor communication within the multi-agency service
2. Difficult geographical access to service
3. Language barriers
4. Parental fear of blame or judgement acting as a barrier to access
5. Lack of health literacy and patient empowerment in Deep End communities
6. Insufficient integration of IT systems between services
7. Financial and spatial limitations

*'Every parent wants the best for their child, so I don’t think they’ll intentionally not engage, but I think there are a lot more barriers for our patients to engage with anything and we need to make it as easy as possible, and it’s not just now in adult mental health services, so if we can make this one more accessible and take away any of those barriers then I think that would be huge.'* (F4)

The new service was viewed as a key opportunity for improved recognition of primary care’s contribution to IMH. One participant emphasised the importance of other services listening to GPs’ concerns to reach the right people at the right time.

It was considered that, for the service to succeed, integration with, or embedding within, PHC would be necessary. Participants suggested that the IMH service should be approached through integration with schools, nurseries, social services, PNMH, CAMHS, adult mental health, and addiction services.

The need for improved HCP training and awareness of IMH was recognised. It was thought that all adult and child health services should be encouraged to consider IMH, where applicable, to maximise outreach and identification of families:

*'It would just have to be like an automatic add-on if someone was joining the service, alcohol or addiction or something, just part of that screening assessment was asking about children in the house, and then identifying them, and then they automatically will be encompassed into that.'* (F7)

Three participants highlighted the importance of co-development with stakeholders, particularly parents from different communities and individuals from relevant professional backgrounds. One participant with experience of service development underlined the need to adopt a pragmatic approach for flexible and efficient coordination of the new service.

Participants raised the possibility of engaging parents by involving them in service delivery through parent-led support groups. Community link workers were considered as having the potential to bridge the gap between communities and IMH services. HVs were identified as best placed to recognise and refer vulnerable families.

## Discussion

### Summary

This study has demonstrated that GPs working in deprived areas have a deep understanding of the issues affecting infant mental health, although do not necessarily relate to the term ‘infant mental health (IMH)’. To increase healthcare practitioner confidence, specific IMH training is needed. Fear of blame, criticism, and social work involvement were seen by GPs as barriers to parental engagement with an IMH service in deprived areas. Participants believed that this could be overcome by considering how the service is presented to communities.

Importantly, this study has highlighted that while new consolidated IMH services are much-needed, particularly in areas of socioeconomic disadvantage, the invaluable pre-existing role of GPs and PHC teams in IMH must be recognised, supported, and integrated into the service. Participants’ unique perspectives, as generalists, underlined the need for any new service to take a holistic approach to IMH, supporting the whole household, to successfully address the needs of infants.

### Strengths and limitations

This is the first qualitative study that the authors are aware of to specifically explore GPs’ views and understanding of IMH. That these GPs were sampled from practices in the most deprived areas — where IMH needs are greatest — is a particular strength of this study.

A potential limitation is that 10 of the 12 participants were women; however, this is in keeping with the demographics of the Deep End Steering Group. It is also important to note that the Deep End Steering Group consists of the most engaged GPs in the Deep End network. As previous research has shown, increased involvement in the Deep End group is associated with a greater understanding of social determinants of health.^35^ A further limitation is that, owing to difficulties recruiting participants from NHSGGC, the final two participants were GPs currently working outside NHSGGC.

### Comparison with existing literature

Qualitative research with a Scottish IMH stakeholder group by Weaver *et al* ^29^ identified key barriers and enablers to IMH service development. Their findings resonate with this study; '*societal stigma and lack of understanding*' and '*lack of synergy*' were common barriers. In the present study, GPs acknowledged the potential role of stigma but focused more on parental fear of blame.

Most IMH research has taken place in high-income countries and, as such, findings may not be transferrable to other cultural, economic, and social contexts.^36,37^ There are, however, some international examples of integration of IMH into primary care. In South Africa, for instance, a screening tool has been implemented in postnatal checks, as well as parental education of positive infant–caregiver interactions through home-visiting, and the use of the Ububele Baby Mat in primary care clinics.^36,38–41^

In the UK, the Leeds IMH service^42^ operates at the following three levels: universal, targeted, and specialist/specialist plus. The universal level is community-based IMH provision for all families across Leeds, and is integrated with midwifery and health visiting, to identify and support vulnerable families. This is in keeping with views of participants in this study, who highlighted the need for HV involvement in the new NHSGGC service. The Leeds universal service has also developed resources for parents and IMH training for HCPs. The targeted level of the IMH services can be referred into by a range of practitioners, notably midwives, HVs, and GPs.

Several participants highlighted the key role that charitable organisations, for example, Home-Start,^43^ play in supporting infants and their family. It is important that IMH services are aware of these activities and can work alongside them, to maximise reach and share resources. Since not all children who have experienced ACEs develop mental health problems, the authors of the present study would not recommend interventions specifically for ACEs; however, there is ongoing research in the group that is currently evaluating an IMH service specifically for children coming into foster care (ClinicalTrials.gov Identifier: NCT01485510), which will report in 2024.

### Implications for research and practice

This study has demonstrated the importance of GP involvement in developing and delivering IMH services. Given current NHS resource limitations, any new service delivery will likely look different to the ideal model discussed by stakeholders. However, a priority highlighted by this research is improving communication between primary and secondary care, in pre-existing as well as new services.

The need for training of primary HCPs to improve understanding of IMH and identification of families who may benefit from additional support has also been identified. In 2019, NHS Education Scotland developed IMH training^44^ that has not been accessed by many GPs despite reaching 18 000 other HCPs in person or online. It is important that GPs are made aware of — and have time to access — such training opportunities. IMH education could also be incorporated into undergraduate and postgraduate curricula.

Future research could explore views of IMH among GPs working in other areas and evaluate access to, and outcomes from, new IMH services. Qualitative research involving parents living in deprived areas is also required to fully understand their perceptions of IMH and their potential use of related services.

## Data

The dataset relied on in this article is available from the corresponding author on reasonable request.
